# Global trends and research hotspots in autoimmune encephalitis: insights from a bibliometric and visualized analysis: 1971-2025

**DOI:** 10.3389/fimmu.2026.1798782

**Published:** 2026-04-22

**Authors:** Yunsheng Liu, Rongde Zhong, Xianlin Wu, Yuyan Wang, Jinfang Zhang, Zengwei Kou

**Affiliations:** 1 Cancer Center, Shenzhen Hospital (Futian) of Guangzhou University of Chinese Medicine, Shenzhen, China; 2 Department of Neurosurgery, Institute of Translational Medicine, Shenzhen Second People’s Hospital/the First Affiliated Hospital of Shenzhen University Health Science Center, Shenzhen, China; 3 College of Basic Medicine and Forensic Medicine, Henan University of Science and Technology, Luoyang, China; 4 Department of Laboratory Medicine and Pathobiology, Temerty Faculty of Medicine, University of Toronto, Toronto, ON, Canada

**Keywords:** artificial intelligence, autoimmune encephalitis, bibliometric analysis, immunotherapy, NMDA receptor, visual analysis

## Abstract

**Background:**

Autoimmune encephalitis (AE) encompasses a highly heterogeneous spectrum of severe immune-mediated neurological disorders. Over the past 50 years, research has expanded rapidly, yet a quantitative synthesis of its evolution, key contributors, and thematic trends is lacking. Here, we provide a comprehensive 50−year, multi−database bibliometric study that systematically maps the full trajectory of AE research-from early descriptions to the current era of mechanism−driven therapeutics-using advanced analytical tools.

**Methods:**

We systematically retrieved AE-related publications from 1971 to May 24, 2025 from PubMed, Web of Science, and Scopus. After deduplication, records were analyzed using bibliometric tools (CiteSpace, VOSviewer, Bibliometrix). Analyses included publication trends, national/institutional contributions, author networks, journal distributions, co-citation patterns, keyword co-occurrence, and thematic evolution.

**Results:**

Annual publications surged after 2007, coinciding with the discovery of anti-NMDA receptor encephalitis (anti-NMDAR AE), reaching a peak in 2022. The USA and China were the leading contributors by volume, while Spain had the highest average citation impact. The University of Pennsylvania and the University of Oxford emerged as the most productive institutions. Dr. Josep Dalmau is the most prolific author in the field, leading a major collaborative cluster. Frontiers in Neurology published the most papers, while Neurology had the highest H-index. Keyword and thematic analyses confirmed anti-NMDAR AE as the dominant research focus, with intense scholarly interest in its pathogenesis, diagnosis, and immunotherapy. Recent trends highlight emerging topics like COVID-19-associated AE, anti-IGLON5 disease.

**Conclusions:**

This study outlines the development, collaboration patterns, and research hotspots in AE. Research continues to center on anti-NMDAR encephalitis, with a growing focus on clinical and therapeutic applications. Future directions include mechanistic studies, biomarker discovery, and advanced immunotherapies.

## 1 Introduction

Autoimmune encephalitis (AE) is a group of severe inflammatory brain diseases mediated by the immune system’s production of autoantibodies targeting neuronal surface antigens (approximately 60% of cases) or intracellular neuronal proteins (1). These immune responses lead to a range of neuropsychiatric symptoms, including changes in mental status, cognitive impairment, and seizure (2). The point prevalence of autoimmune encephalitis was estimated to be 13.7 per 100,000 people as of January 1, 2014, based on a population-based study in Olmsted County, Minnesota (3).

More than 20 subtypes of AE have been identified, with some of the most well-characterized forms including anti-N-methyl-D-aspartate receptor (NMDAR) encephalitis (4), anti-leucine-rich glioma-inactivated 1 encephalitis (5), and anti-myelin oligodendrocyte glycoprotein (MOG) antibody-associated disorders (6). Of these, autoimmune encephalitides targeting ionotropic glutamate receptors-such as anti-NMDA receptor (4, 7), anti-AMPA receptor (8), and anti-KA receptor encephalitis (9)-have been particularly well-studied in terms of their pathogenic mechanisms and treatment approaches. Pathogenically, these conditions are primarily driven by autoantibodies that bind to their respective synaptic receptors. In anti-NMDA receptor encephalitis, antibodies induce cross-linking and internalization of NMDA receptors, a critical ligand-gated calcium channel in the brain (10, 11), resulting in disrupted glutamatergic signaling, impaired synaptic plasticity, and widespread network dysfunction. Clinically, this catastrophic receptor failure manifests as severe neuropsychiatric symptoms, intractable seizures, and profound autonomic instability. By analogy with anti-NMDAR encephalitis, we propose that anti-AMPA and anti-KA receptor encephalitis are also driven by antibody-mediated internalization of surface receptors, resulting in impaired excitatory synaptic transmission and neuronal hyperexcitability. Therapeutically, first-line management for all subtypes typically involves immunotherapy-corticosteroids, intravenous immunoglobulin (IVIG), and/or plasma exchange (PLEX), to rapidly lower pathogenic antibody levels. When a tumor is identified, such as ovarian teratoma in anti-NMDA receptor encephalitis, prompt resection is critical and often leads to marked clinical improvement. Patients who do not respond adequately to first-line treatments are usually escalated to second-line agents such as rituximab (an anti-CD20 monoclonal antibody) or cyclophosphamide to achieve sustained B-cell depletion and immunosuppression. Throughout the course of illness, supportive care and symptomatic management-including antiepileptic drugs for seizure control and antipsychotics for behavioral disturbances-remain essential components of comprehensive care. The triggers of AE are not yet fully understood. Available evidence suggests that viral infections, such as herpes simplex virus encephalitis, may initiate autoimmune responses in some patients (12). Others may develop AE in response to novel cancer immunotherapies, particularly immune checkpoint inhibitors, or in the context of malignancies such as teratomas. Importantly, a substantial proportion of AE cases present without any identifiable trigger or predisposing factor, with some studies reporting that 32% of patients have unidentified triggers (13).

Clinically, AE can manifest as diffuse encephalopathy or localized dysfunction of the cerebellar or brainstem regions. Common symptoms include behavioral changes, psychosis, memory and cognitive deficits, seizures, and disturbances in sleep (1). Diagnostic workups typically involve the assessment of age- and sex-specific patterns, neuroimaging, detection of neural autoantibodies, and cerebrospinal fluid analysis (14). However, relapse occurs in approximately 10-30% of anti-NMDAR AE patients within two years after the initial episode (3). Over the past five decades, significant progress has been made in understanding AE’s pathogenesis, classification, diagnostic tools, and therapeutic strategies. The volume of AE-related research has grown rapidly, encompassing molecular mechanisms, clinical phenotyping, treatment innovations, and case reports. However, this growing body of literature is highly heterogeneous and scattered across disciplines, making it challenging for researchers to gain a comprehensive understanding of the field’s evolution and current landscape.

Bibliometric analysis serves as a robust approach for systematically surveying extensive and intricate bodies of literature (7). By quantitatively examining publication characteristics, such as author networks, institutional affiliations, national contributions, subject categories, and keyword trends, researchers can map the development of a field, identify influential contributors, and detect emerging hotspots, and uncover hidden collaborative patterns that might otherwise remain obscured. In this study, we employed bibliometric methodologies to analyze a comprehensive dataset of AE-related literature from the PubMed, Web of Science, and Scopus. PubMed was selected for its comprehensive coverage of biomedical and life sciences literature, particularly its strength in indexing clinical and preclinical studies relevant to neurology and immunology. Web of Science was included for its robust citation indexing across scientific disciplines and its long-standing use in bibliometric research. Scopus was chosen to complement these sources with broader coverage of interdisciplinary and emerging research, including relevant publications in fields such as pharmacology and computational biology. Although there is some overlap among these databases, this multi-source approach minimizes database-specific biases (e.g., variations in journal coverage, indexing practices, and geographic representation) and enhances the completeness of our literature sample, thereby providing a more reliable foundation for bibliometric analysis.

This study maps the AE research landscape by highlighting key contributors, collaboration patterns, publication trends, and thematic evolution. These insights provide a valuable reference for clinicians and scientists, enabling a more targeted and strategic approach to future AE research.

## 2 Methods and materials

This study was conducted without the use of animal studies or human participants, thus an ethics approval was not applicable.

### 2.1 Data retrieval and collection

On May 24, 2025, we utilized the Web of Science, PubMed, and Scopus databases for data collection, as previously reported (15, 16). Web of Science is a premier research platform that provides comprehensive information across the sciences, social sciences, arts, and humanities. It is recognized as an independent global citation database by one of the world’s most trusted publishers. PubMed, on the other hand, is extensively accessed in the medical field and is among the most frequently used databases by healthcare professionals and researchers. Scopus is also a worldwide scientific abstract and citation database launched by the academic publisher Elsevier.

For the Web of Science, we employed the following search query:

TS=(“autoimmune encephalitis” OR “autoimmune encephalitides” OR “antibody mediated encephalitis” OR “anti-NMDAR encephalitis” OR “anti-LGI1 encephalitis” OR “anti-GABABR encephalitis” OR “anti-AMPAR encephalitis” OR “anti-CASPR2 encephalitis” OR “anti-MOG encephalitis” OR “anti-Hu encephalitis” OR “anti-Yo encephalitis” OR “anti-Ri encephalitis” OR “anti-Ma2 encephalitis” OR “anti-CV2 encephalitis” OR “anti-GAD encephalitis” OR “NMDA receptor encephalitis” OR “LGI1 antibody encephalitis” OR “CASPR2 antibody encephalitis” OR “GABA-B receptor encephalitis” OR “AMPAR antibody encephalitis”).

The “TS” field denotes a “Topic Search”, which includes the Title, Abstract, and Keywords.

For the PubMed, we employed the following search query:

“autoimmune encephalitis”[Title/Abstract] OR “autoimmune encephalitides”[Title/Abstract] OR “antibody mediated encephalitis”[Title/Abstract] OR “anti-NMDAR encephalitis”[Title/Abstract] OR “anti-LGI1 encephalitis”[Title/Abstract] OR “anti-GABABR encephalitis”[Title/Abstract] OR “anti-AMPAR encephalitis”[Title/Abstract] OR “anti-CASPR2 encephalitis”[Title/Abstract] OR “anti-MOG encephalitis”[Title/Abstract] OR “anti-Hu encephalitis”[Title/Abstract] OR “anti-Yo encephalitis”[Title/Abstract] OR “anti-Ri encephalitis”[Title/Abstract] OR “anti-Ma2 encephalitis”[Title/Abstract] OR “anti-CV2 encephalitis”[Title/Abstract] OR “anti-GAD encephalitis”[Title/Abstract] OR “NMDA receptor encephalitis”[Title/Abstract] OR “LGI1 antibody encephalitis”[Title/Abstract] OR “CASPR2 antibody encephalitis”[Title/Abstract] OR “GABA-B receptor encephalitis”[Title/Abstract] OR “AMPAR antibody encephalitis”[Title/Abstract] OR (“Encephalitis, Autoimmune”[MeSH]).

PubMed does not have a separate search field for Keywords.

For the Scopus, we employed the following search query:

TITLE-ABS-KEY(“autoimmune encephalitis” OR “autoimmune encephalitides” OR “antibody mediated encephalitis” OR “anti-NMDAR encephalitis” OR “anti-LGI1 encephalitis” OR “anti-GABABR encephalitis” OR “anti-AMPAR encephalitis” OR “anti-CASPR2 encephalitis” OR “anti-MOG encephalitis” OR “anti-Hu encephalitis” OR “anti-Yo encephalitis” OR “anti-Ri encephalitis” OR “anti-Ma2 encephalitis” OR “anti-CV2 encephalitis” OR “anti-GAD encephalitis” OR “NMDA receptor encephalitis” OR “LGI1 antibody encephalitis” OR “CASPR2 antibody encephalitis” OR “GABA-B receptor encephalitis” OR “AMPAR antibody encephalitis”).

### 2.2 Data analysis

The PubMed records were initially obtained in.nbib format, Web of Science in plain text (.txt), and Scopus in CSV (.csv). To standardize the data, PubMed and Scopus files were first converted into Web of Science plain text format using the format conversion tool embedded in CiteSpace (V6.4.R1). Subsequently, all datasets in this unified format were concatenated into a single file with the Linux cat command and imported into CiteSpace for duplicate detection and removal. Since the.txt files exported from CiteSpace are in a general format accepted by all subsequent analysis software, no further format conversion or information extraction was required thereafter. In brief, duplicates were identified based on the first author’s last name, the first 10 letters of the title, the first 10 letters of the source, the publication year, the DOI (if available), and the first page number. To further validate the deduplication process, we randomly selected 10 publications using NotePad++ and manually checked for duplicates; no duplicate records were found. The cleaned dataset was then re-exported in Web of Science plain text format, merged again if needed, and finally used as the standardized input for analyses conducted with CiteSpace, VOSviewer (1.6.20) and Bibliometrix (5.1.0). To ensure the objectivity and comprehensiveness of the analysis, we systematically excluded retracted papers, corrections, and news items from the databases. It is worth noting that during this process, we did not impose any language restrictions or document type restrictions (except news items). To further validate the relevance of the retrieved literature, each author independently reviewed 50 randomly selected publications, and no irrelevant papers were identified; therefore, no further manual filtering was applied.

To enhance the transparency and verifiability of the data presented in this study, we have publicly archived the following materials on the Zenodo repository (Accession Number: 10.5281/zenodo.18310832): the raw data files downloaded from PubMed (.nbib), Web of Science (.txt), and Scopus (.csv), as well as the standardized and de-duplicated dataset generated by CiteSpace. This cleaned dataset corresponds to the version used in all subsequent analyses reported in this study. These files are available for interested researchers to access, review, and independently verify our data processing workflow and subsequent analyses.

For the comparison and visualization of differences in author count, citation count, and citation frequency between Review Articles and Research Articles, we performed the analyses using GraphPad Prism (version 8.0). Prior to analysis, the normality of the data was assessed using the Shapiro-Wilk test. A natural logarithmic transformation was applied to achieve approximate normality. Homogeneity of variances was evaluated using Levene’s test. Quantitative comparisons between the two groups were conducted using two-tailed Student’s t-tests, with P values indicated directly in the figures.

## 3 Results

### 3.1 Annual and cumulative publication trends

**Figure 1:**
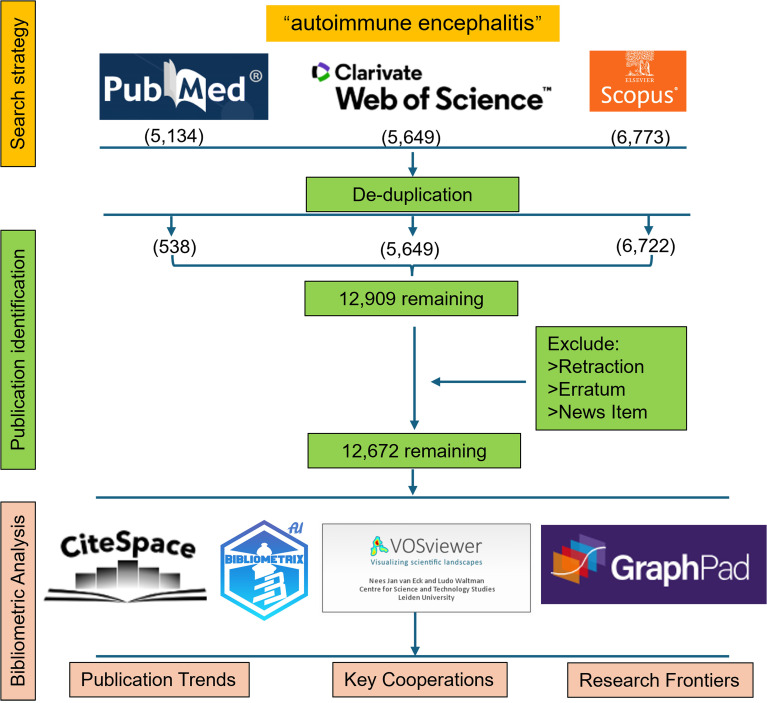
Flow chart of literature search and screening. Duplicate removal was performed using CiteSpace with default parameters. Retractions and errata were identified by searching the title (TI) field in NotePad++ for the terms “retraction” and “erratum”, and records containing these terms were excluded. News items were excluded by searching for the document type (DT) field in NotePad++.

**Figure 2:**
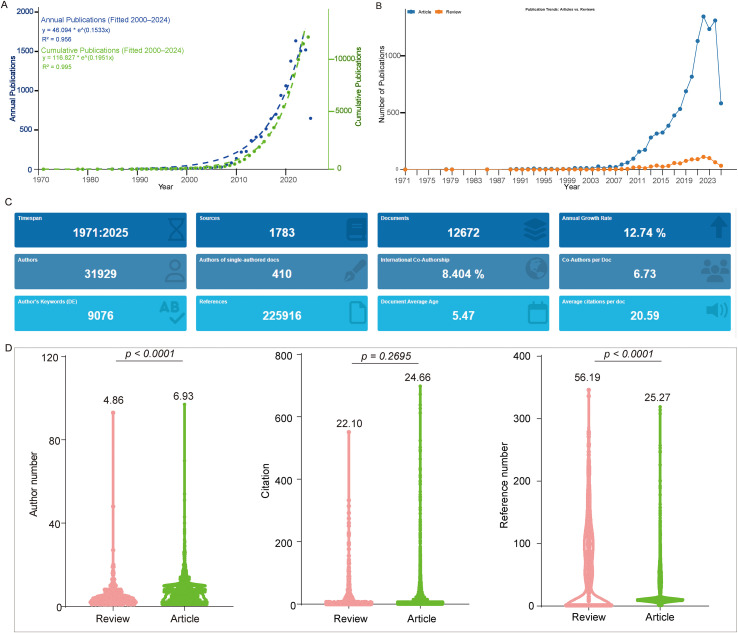
Analysis of trends and characteristics of publications. **(A)** Temporal trends in autoimmune encephalitis (AE) research output, showing annual publication counts and cumulative publication volume. Data was extracted using a customized R package based on Bibliometrix. **(B)** Publication trends of original articles and reviews in AE research, generated by extracting document type (DT) fields (“article” and “review”) using the same customized R package. **(C)** Bibliometric summary of publication characteristics, generated by Biblioshiny under default settings. **(D)** Comparison between reviews and original articles based on author count (AU), total citations (TC), and reference count (CR). Data were extracted using a customized R package based on Bibliometrix. Statistical comparisons were performed using appropriate methods (as detailed in the Methods section).

We retrieved 5,134 publications from PubMed, 5,649 from Web of Science (WOS), and 6,773 from Scopus. Using CiteSpace’s format conversion feature, we converted the PubMed and Scopus datasets into the WOS format and merged them. This resulted in 12,909 records being recognized after deduplication. After excluding retraction articles, corrections, and news items, 12,672 publications were obtained (**Figure 1**). From 1971 to 2000, the cumulative number of publications remained below 100, suggesting slow initial development in the field. Annual publications only exceeded 100 per year in 2010, indicating a possible breakthrough around that time. To quantitatively characterize this growth pattern, we fitted exponential growth models to publication data from 2000 to 2024. The results confirmed a strong exponential fit for both cumulative and annual publications, with the following equations: for annual publications y = 49.094 × e^(0.1533x) (R² = 0.956) and for cumulative publications y = 116.827 × e^(0.1951x) (R² = 0.995). Annual publications reached a peak of 1,634 in 2022, followed by 1,505 in 2023 and 1,518 in 2024. As of May 24, 2025, a total of 648 papers had already been published in 2025 (**Figure 2A**), indicating that research activity in this field remains robust.

Publications were authored by 31,929 individuals and appeared across 1,783 journals, averaging 6.73 authors per article, which reflects both the complexity of the research and the collaborative nature of this field. The average number of citations per article, including self-citations and without normalization, was 20.59 (**Figure 2C**). To further analyze the publication patterns of different article types, we extracted data for Articles and Reviews. We found that since 2000, the number of Articles has consistently far exceeded that of Reviews. This difference widened in 2007 and became even more pronounced in 2013 (**Figure 2B**). Additional analysis revealed that Reviews generally involved fewer authors than Articles but cited substantially more references. In terms of received citations, no significant differences were observed between the two types, although Articles had a slightly higher average citation count, exceeding Reviews by 2.56 citations (**Figure 2D**).

### 3.2 Countries and regions with the highest publication output

**Figure 3:**
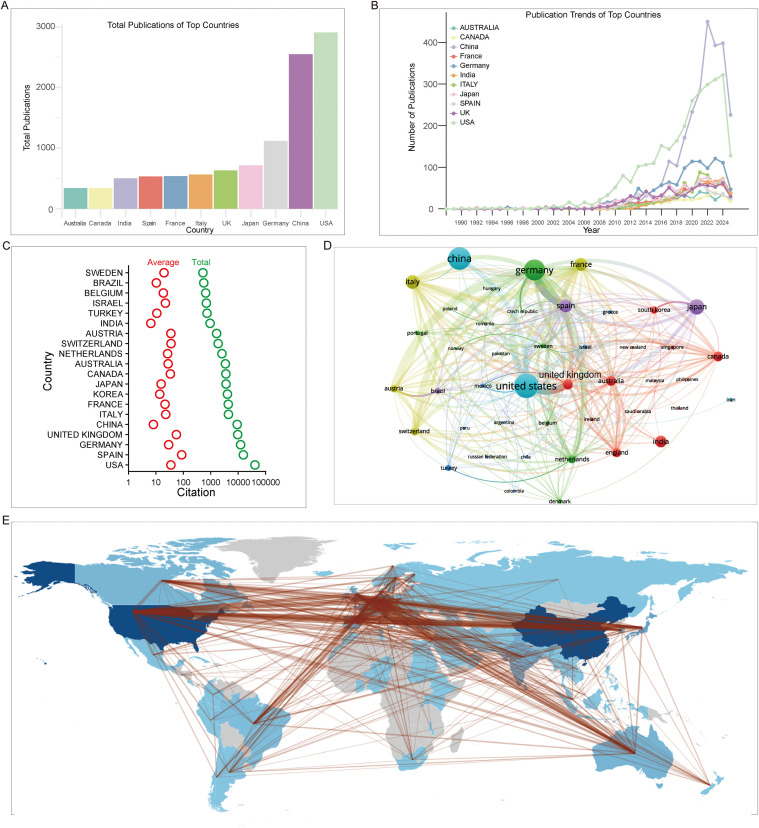
National contributions and collaboration patterns in AE research. **(A)** Top countries ranked by the number of corresponding authors, extracted from the C1 field using a customized R package based on Bibliometrix. **(B)** Temporal trends of the top 11 most productive countries in AE research, generated by plotting publication counts against year using the same customized R package. **(C)** Top 20 countries with the highest average and total citations, produced by Biblioshiny under default settings. **(D)** Overlay network visualization of countries engaged in AE research, created with VOSviewer based on document co−authorship analysis (default parameters). **(E)** Global collaboration map showing country−level research output and regional partnerships, also generated by Biblioshiny under default settings.

Based on the total number of publications, the United States and China are the top two contributors, each having published over 2,000 papers. These two countries significantly outpaced the others in the top ten. Notably, any country with more than 500 publications would rank within the top eight globally (**Figure 3A**). In terms of publication trends, leading countries generally entered a phase of accelerated growth around 2007. Among them, the United States stands out as the most prominent, having since pulled far ahead of other nations. We observed that China, the second-highest contributor, experienced a rapid surge in publications around 2016, nearly exponential in growth, peaking in 2022 before showing a slight decline in 2023 and 2024. Other top countries exhibited relatively stable growth compared to the U.S. and China (**Figure 3B**). The average international collaboration rate-defined as the proportion of publications co−authored by authors from at least two different countries-was 9.0%. Among the top ten countries, the USA, Germany, Japan, France, Italy, Spain, and the UK exceeded this average, while China, South Korea, and especially India fell below it. India, in particular, showed an international collaboration rate of only 2%, suggesting that emerging research nations in South and East Asia may need further development to establish broader collaborative networks (data not shown).

In terms of total citations, the USA, Spain, Germany, and the UK were the leaders. While the USA had the most total citations, Spain had the highest average citations per article at 86.8, surpassing the USA’s average. In contrast, China and India had lower-than-average citation rates. As these countries publish follow-up studies, they also contribute to the citations of earlier foundational work (**Figure 3C**). The citation burst analysis not only identifies traditionally high-impact countries but also highlights emerging citation activity. Burst strength, as calculated by CiteSpace, quantifies the intensity of a sudden increase in citation frequency over a specific time interval, indicating periods of particularly high scholarly interest. The USA maintained strong citation bursts between 1982 and 2011. Remarkably, Israel sustained a high citation activity from 1993 to 2010. Japan had the highest citation burst strength (26.81), followed by Spain (24.61). More recently, Iran, Turkey (Türkiye), and Russia have shown an increase in citation strength (**Table 1**).

**Table 1:** Top 20 countries with the strongest citation bursts.

Countries	Year	Strength	Begin	End	1971 - 2025
USA	1982	18.21	1982	2011	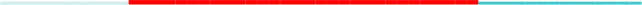
Israel	1993	8.49	1993	2010	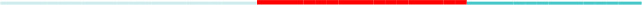
Australia	1993	5.4	1993	2004	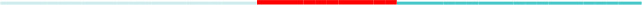
Switzerland	1987	19.23	2000	2012	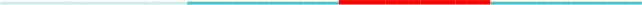
Japan	1990	26.81	2006	2012	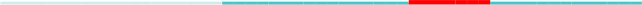
England	1978	21.45	2008	2017	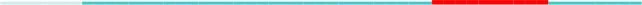
Germany	1994	4.11	2008	2009	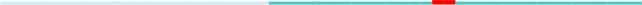
Canada	1994	8.8	2010	2011	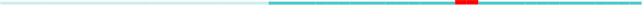
Hungary	2011	6.67	2011	2016	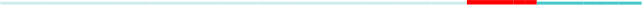
Ireland	2008	6.18	2011	2013	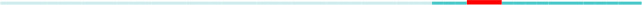
Spain	1990	24.61	2012	2015	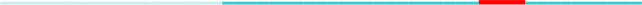
UK	1978	7.76	2012	2014	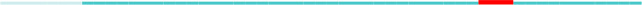
Czech Republic	2001	6.44	2013	2018	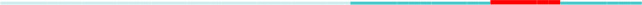
Turkey	2012	6.04	2013	2014	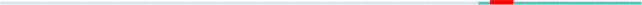
Sweden	1989	7.2	2014	2017	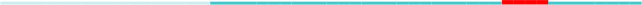
Bulgaria	2018	4.48	2018	2020	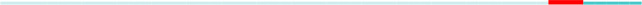
Thailand	2013	4.26	2018	2019	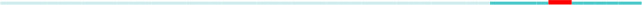
Iran	2006	4.21	2022	2023	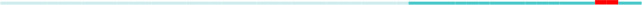
Türkiye	2023	6.51	2023	2025	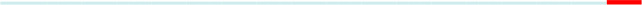
Russia	2008	4.24	2023	2025	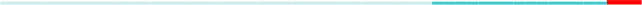

The analysis of the international collaboration network revealed the formation of several distinct clusters. The largest and most prominent cluster is centered on the United States, encompassing collaborative partners such as the United Kingdom, Mexico, and Sweden. Alongside this primary hub, other significant clusters have formed, centered on China, Germany, Canada, India, Italy, and the Netherlands, respectively (**Figure 3D**). A geographic visualization of these collaborations shows a dense and interconnected network primarily concentrated in Europe, with strong extensions to North America, Australia, and East Asia (**Figure 3E**).

### 3.3 Institutional and journal publication trends

**Figure 4:**
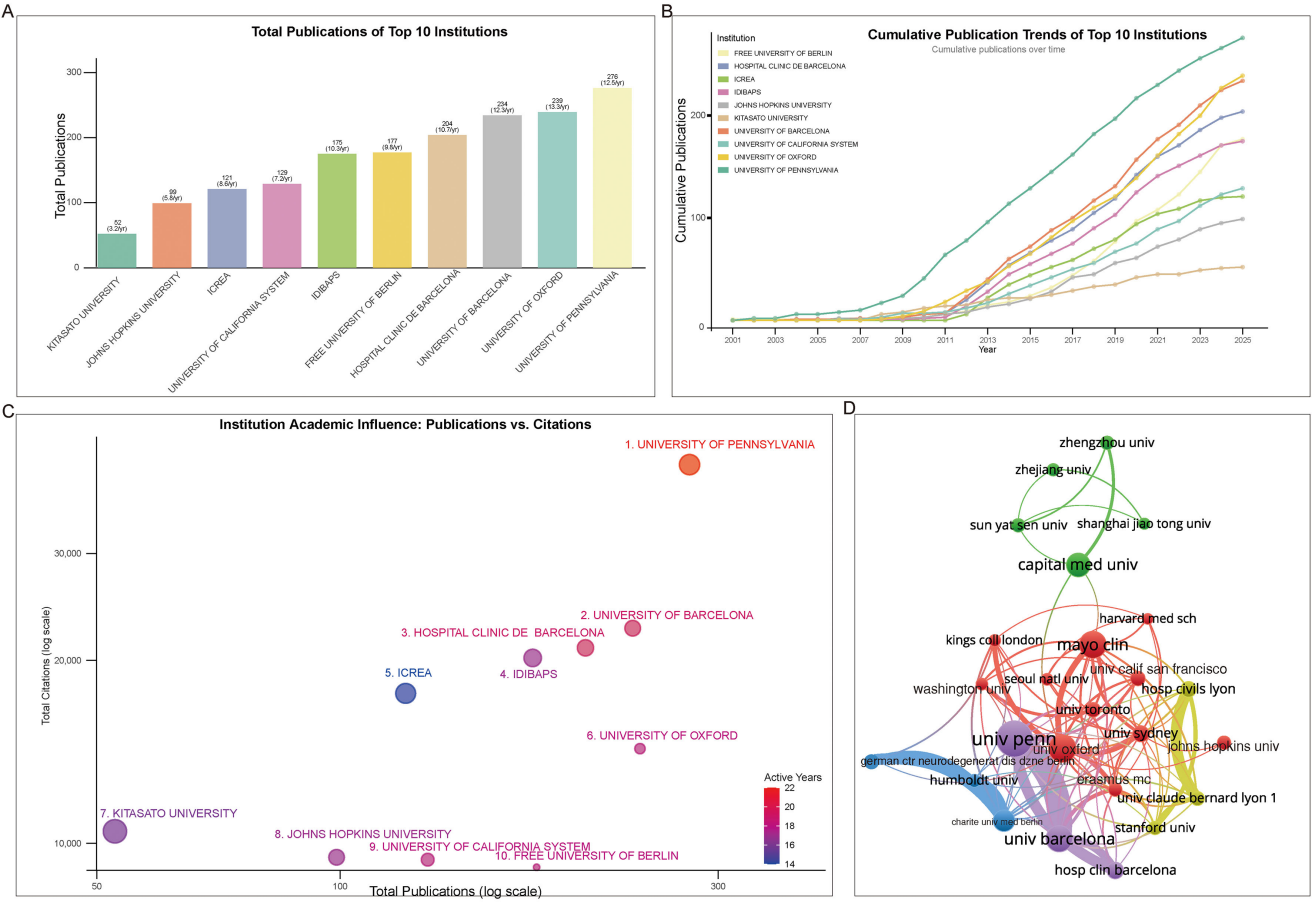
Trends in author affiliations engaged in AE research. **(A)** Total publication output of the top universities and research institutions in autoimmune encephalitis (AE) research. Data was extracted from the C1 field using a customized R package based on Bibliometrix. **(B)** Temporal trends of the most productive institutions in AE research, generated by plotting publication counts against year using the same customized R package. **(C)** Scatter plot of representative institutions engaged in AE research, showing the relationship between total publications and total citations. Citation data were extracted via a customized R script based on Bibliometrix. **(D)** Overlay network visualization of institutional collaboration, created with VOSviewer (weights by documents) based on co−authorship relationships among institutions.

Based on the total number of publications, the University of Pennsylvania ranks first with a substantial lead, having contributed 276 papers. Following closely are the University of Oxford and the University of Barcelona, each with over 230 publications. All leading institutions in the analysis have published more than 52 papers (**Figure 4A**). A temporal analysis of publication trends for selected representative institutions shows that the University of Pennsylvania surged to the top around 2003, followed by a period of rapid growth. Other institutions subsequently entered similar expansion phases. Notably, the University of Barcelona experienced the most rapid rise within the shortest timeframe, surpassing its peers between 2011 and 2024 to become a leading institution in this field (**Figure 4B**). When both publication volume and total citations are considered, the University of Pennsylvania remains in first place, while the University of Barcelona rises to second, followed by the Hospital Clínic de Barcelona (**Figure 4C**). The institutional collaboration network reveals two spherical clusters. The larger cluster centers on leading institutions in Europe and North America, including the University of Pennsylvania, University of Oxford, University of Toronto, and University of Barcelona, among others, demonstrating dense and diverse collaborative ties. The smaller cluster is primarily composed of Chinese institutions such as Capital Medical University, Shanghai Jiao Tong University, and Sun Yat-sen University, with Capital Medical University serving as a key bridge linking this cluster to the larger network through collaborations with institutions like the University of Oxford and the Mayo Clinic (**Figure 4D**).

Citation burst analysis indicates that the University of Pennsylvania had the strongest burst strength (53.42), followed by the University of Barcelona, with both institutions showing similar periods of activity. More recently, Chinese institutions, including Zhengzhou University and its affiliated hospital, Zhejiang University, Shanghai Jiao Tong University, and French University Claude Bernard Lyon, have emerged as active contributors (**Table 2**).

**Table 2:** Top 25 institutions with the strongest citation bursts.

Institutions	Year	Strength	Begin	End	1971 - 2025
University of Geneva	2000	12.66	2000	2012	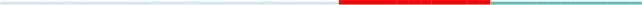
University of Pennsylvania	2002	53.42	2004	2015	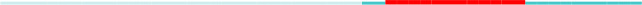
Kitasato University	2008	7.85	2008	2014	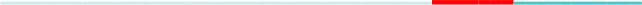
Kanazawa Medical University	2010	11.07	2010	2015	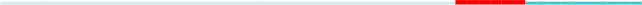
The Children's Hospital at Westmead	2010	10.78	2010	2017	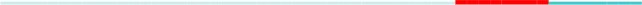
University of Sydney	2010	9.09	2010	2017	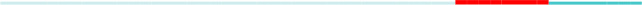
EUROIMMUN	2010	8.53	2010	2017	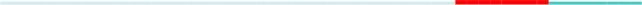
NSW Health	2010	7.14	2010	2017	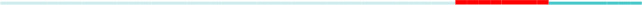
University of Barcelona	2010	20.21	2012	2016	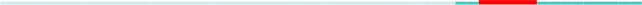
Hospital Clinic de Barcelona	2004	17.23	2012	2015	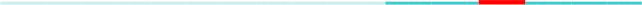
ICREA	2012	16.57	2012	2016	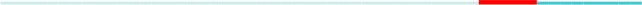
IDIBAPS	2004	15.17	2012	2015	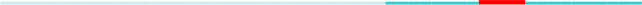
Universite Jean Monnet	2012	7.67	2012	2016	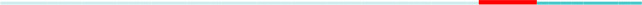
Istanbul University	2013	9.9	2013	2017	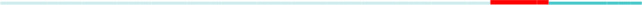
CNRS - National Institute for Biology (INSB)	2011	7.1	2016	2020	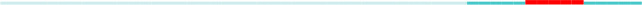
VA Boston Healthcare System	2018	7.35	2018	2019	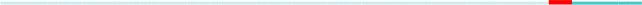
The First Affiliated Hospital of Zhengzhou University	2021	7.33	2021	2023	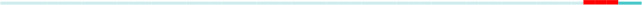
Tehran University of Medical Sciences	2019	7.17	2021	2023	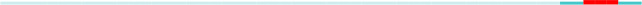
Neurology	2021	9.6	2022	2025	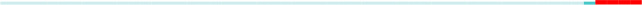
Western University	2022	8.91	2022	2025	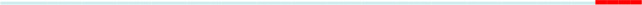
Zhejiang University	2016	8.81	2022	2023	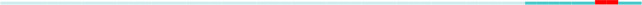
Internal Medicine	2020	8.6	2022	2025	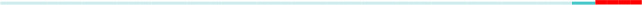
Shanghai Jiao Tong University	2021	7.81	2022	2025	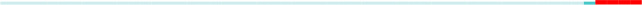
Zhengzhou University	2020	7.5	2022	2025	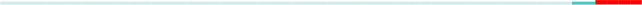
Université Claude Bernard Lyon	2015	8.7	2023	2025	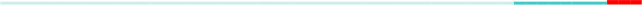

**Figure 5:**
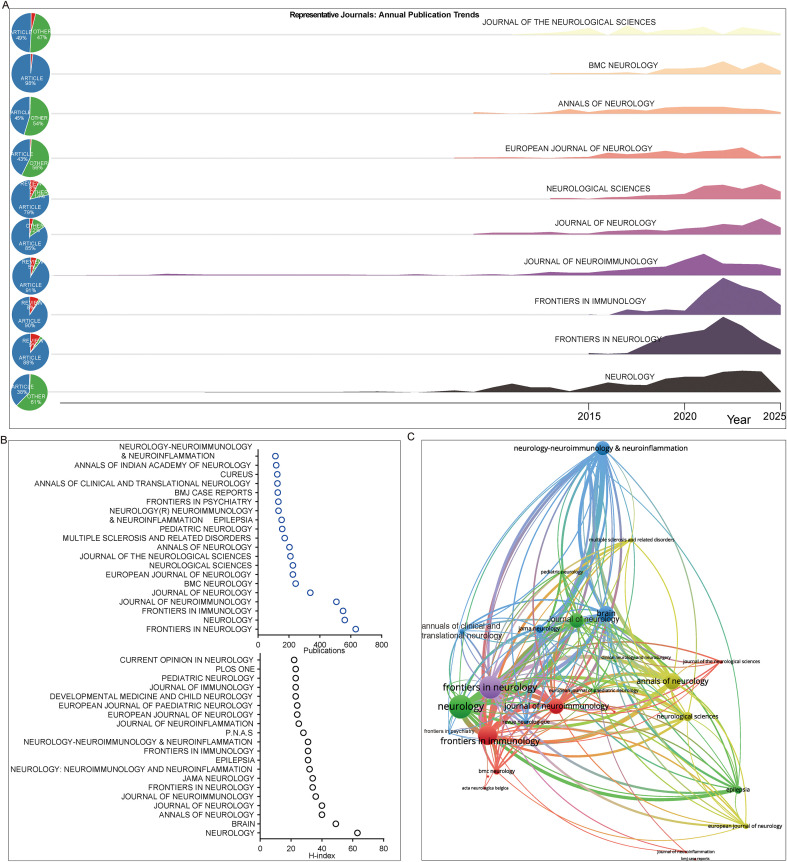
Trends in journals that engaged in AE research. **(A)** Temporal trends of representative journals in AE research, showing annual publication counts. Data were extracted from the SO (source) field using a customized R package based on Bibliometrix and plotted against publication year (PY). Document type distribution within each journal was also derived from the DT field. **(B)** Dot plot of the top 20 most productive and most cited journals in AE research, generated by Biblioshiny under default settings. **(C)** Overlay network visualization of journal co−citation relationships, created with VOSviewer (weights by citations).

We examined publication trends over time in selected journals, including the *Journal of Neuroimmunology*, *Frontiers in Neurology*, *Frontiers in Immunology*, *Neurology*, *Journal of Neurology*, and others. Initially, the *Journal of Neuroimmunology* showed the strongest focus on this field, followed by *Neurology* and the *Journal of Neurology*. As the field gained broader attention, other journals began publishing substantially more research in this area around 2015, most notably *Frontiers in Neurology* and *Frontiers in Immunology.* Among the analyzed journals, most primarily published original research articles (Articles), while some featured other publication types such as case reports and brief communications, rather than reviews (**Figure 5A**).

In terms of publication volume, *Neurology* consistently led initially, followed by the *Journal of Neuroimmunology*. Around 2023, *Frontiers in Neurology* surpassed other journals to become the top publisher in this field and has remained in the lead since (**Figure 5B**). When ranking journals by both publication volume and H-index, *Frontiers in Neurology* ranked first in output but sixth in impact, whereas *Neurology*, which ranked second in output, held the highest H-index (**Figure 5B**).

The journal co-occurrence network formed a large, interconnected cluster, with the *Journal of Neurology*, *Frontiers in Immunology*, and *Frontiers in Neurology* at its core. Other prominent journals included *Neurology, Neurology-Neuroimmunology & Neuroinflammation, and Brain*. Surrounding these central journals were several regionally focused titles, such as the *European Journal of Neurology* and the *Journal of the Neurological Sciences* (**Figure 5C**).

Citation burst analysis of journals revealed that traditional journals such as the *Journal of Immunology* and the *European Journal of Immunology* were active from approximately 1990 to 2015, alongside *Nature* and the *Journal of Experimental Medicine*. *Annals of Neurology* and the *Journal of Neuroscience* showed slightly later activity, around 2008-2018. More recently, journals such as *Frontiers in Immunology* and the *Frontiers in Neurology* have exhibited increased citation bursts (**Table 3**).

**Table 3:** Top 25 cited journals with the strongest citation bursts.

Cited journals	Year	Strength	Begin	End	1971 - 2025
J Immunol	1978	88.01	1978	2014	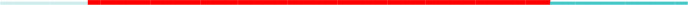
J Exp Med	1978	68.62	1978	2013	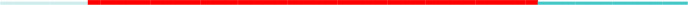
Eur J Immunol	1989	64.47	1989	2015	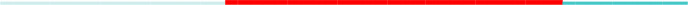
Nature	1989	58.37	1989	2012	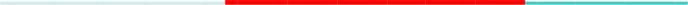
Science	1991	56.36	1991	2012	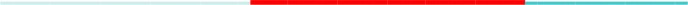
PNAS	1991	51	1991	2012	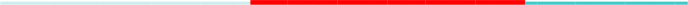
Annals Rev Immunol	1991	46.12	1991	2012	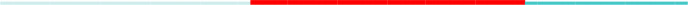
Am J Pathol	1991	39.47	1991	2013	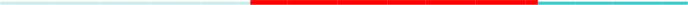
Immunity	1998	39.51	1998	2012	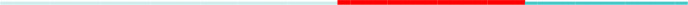
Nat Med	2000	51.73	2000	2012	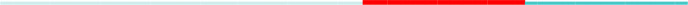
J Child Neurol	1991	42.78	2000	2012	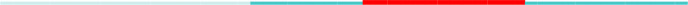
J Biol Chem	2001	44.46	2001	2014	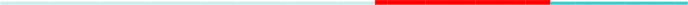
JAMA Neurology	1978	57.02	2004	2016	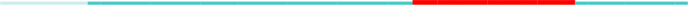
Nature Reviews Neurology	2007	42.88	2007	2016	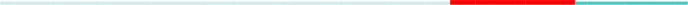
Annals of Neurology	2000	50.51	2008	2014	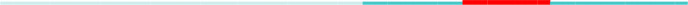
Movement Disord	2004	37.13	2009	2018	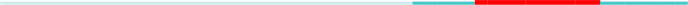
J Neurosci	2004	74.45	2010	2018	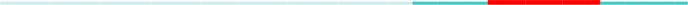
Arch Dis Child	2010	42.64	2010	2018	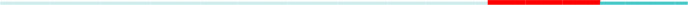
Arch Neurol	1998	41.96	2010	2016	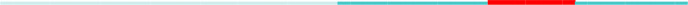
J Pediatr	2010	38.65	2010	2018	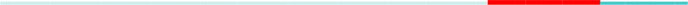
J Child Neurol	2010	42.59	2011	2018	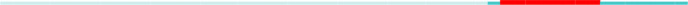
J Pediatr	2010	38.16	2013	2018	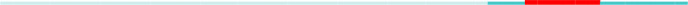
Front Neurol	2015	88.89	2022	2025	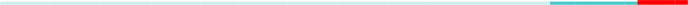
Int J Mol Sci	2018	37.24	2022	2025	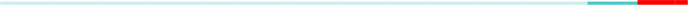
Front Immunol	2016	95.47	2023	2025	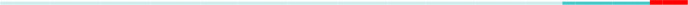

### 3.4 Author publication summary and collaboration patterns

**Figure 6:**
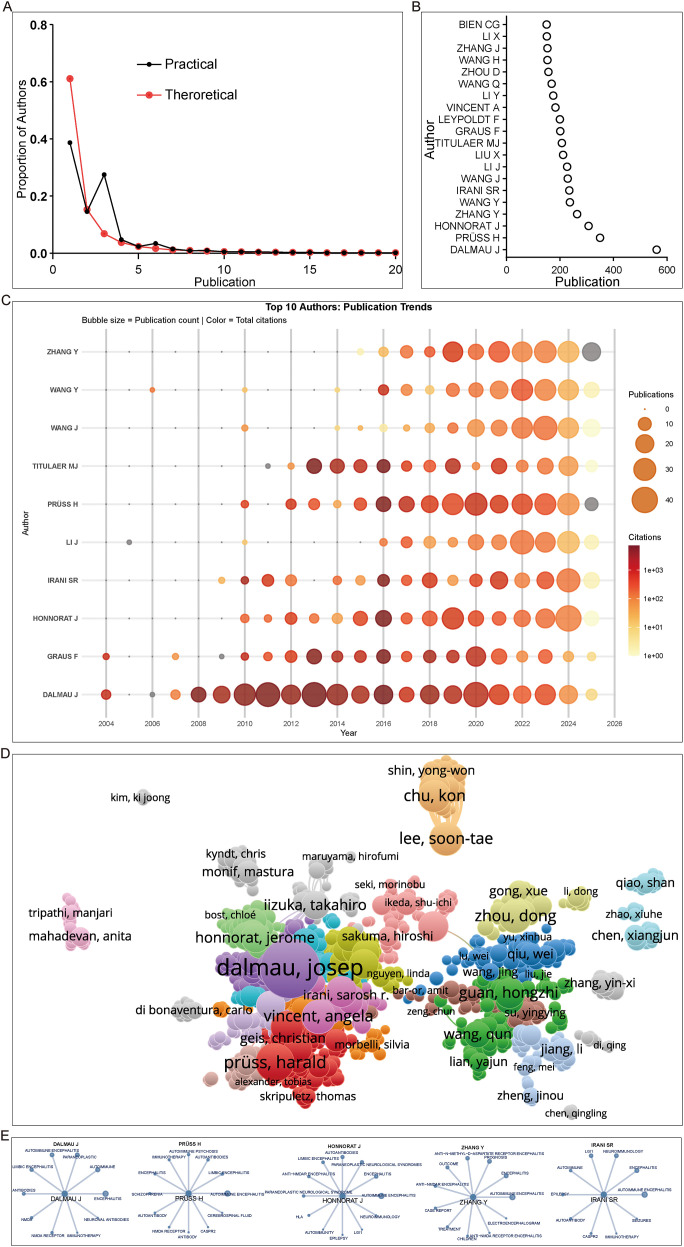
Author summary in AE research. **(A)** Author productivity distribution fitted to Lotka’s law, generated by Biblioshiny under default settings. **(B)** Dot plot of the top 20 most productive authors in AE research, generated by Biblioshiny under default settings. **(C)** Temporal trends of the top 10 most productive authors in AE research, generated by Biblioshiny under default settings. **(D)** Overlay network visualization of author co−citation relationships, created with VOSviewer (weights by documents). **(E)** Keyword profiles of the top five most productive authors. Data were extracted from the AU (author) and DE (author keywords) fields using a customized R package based on Bibliometrix.

Among the over 30,000 contributing authors in the field, approximately 80% have published three or fewer papers (**Figure 6A**). Based on the merged dataset from PubMed, Web of Science, and Scopus after deduplication using full counting, a few prominent scholars stand out for their prolific outputs; For instance, Josep Dalmau has authored 561 publications, far surpassing the other most prolific authors. All of the top 20 authors have contributed more than 150 publications each (**Figure 6B**).

Temporal analysis of publication trends among the top 10 authors revealed that Josep Dalmau has remained consistently active for over two decades, maintaining both high productivity and influence throughout his career. Authors such as Yonghua Wang and Jing Li have also been active for nearly 20 years. In contrast, authors like Harald Prüss and Jérôme Honnorat have been influential for over a decade, while Yuan Zhang has emerged only in the past 10 years but has shown a rapid increase in output in recent years (**Figure 6C**).

The author co-occurrence network revealed two major collaborative clusters. The first cluster centers around Josep Dalmau, with close collaborations involving Jérôme Honnorat, Angela Vincent, and Harald Prüss, the latter forming a secondary hub closely associated with the Dalmau cluster. The second major cluster is centered around Hongzhi Guan, with collaborators including Dong Zhou, Qun Wang, and Yingying Su, indicating a strong Chinese research network. Additionally, Kon Chu and Soon-Tae Lee formed a smaller, independent collaboration cluster peripheral to the two larger hubs (**Figure 6D**). Analysis of keywords from the most influential authors revealed that keywords related to limbic encephalitis and anti-NMDA receptor autoimmunity appeared in the keywords of five authors (**Figure 6E**).

Citation burst analysis of authors revealed that the most cited author FLORANCE NR was active from approximately 2010 to 2017 (strength: 135.07), the authors immediately following are VINCENT A and ABBOUD H, with citation strengths were 115.17 and 108.78, respectively (**Table 4**). This analysis highlights how a vast majority of authors produce minimal output while the field’s direction is shaped by a few prolific, well-connected hubs and their enduring collaborative networks.

**Table 4:** Top 25 cited authors with the strongest citation bursts.

Cited Authors	Year	Strength	Begin	End	1971 - 2025
VINCENT A	2008	115.17	2008	2017	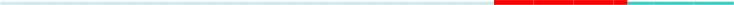
IIZUKA T	2008	101.92	2008	2015	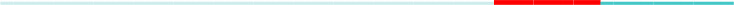
SANSING LH	2008	63.53	2008	2017	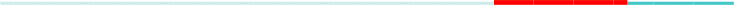
VITALIANI R	2008	55.47	2008	2017	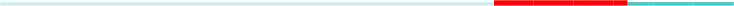
BATALLER L	2008	49.14	2008	2015	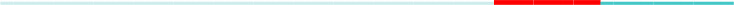
SEKI M	2008	45.04	2008	2017	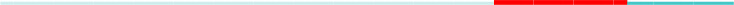
THIEBEN MJ	2008	44.29	2008	2016	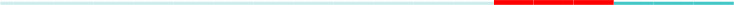
ISHIURA H	2009	50.59	2009	2017	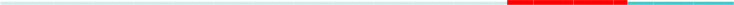
FLORANCE NR	2009	135.07	2010	2017	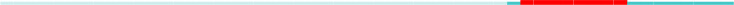
HUGHES EG	2010	63.64	2010	2017	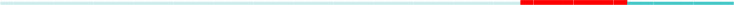
GABLE MS	2010	58.65	2010	2015	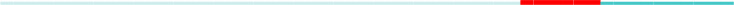
NIEHUSMANN P	2010	40.88	2010	2017	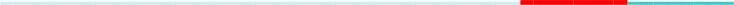
IRANI SR	2010	85.28	2011	2017	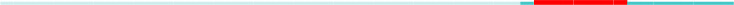
ZANDI MS	2011	45.06	2011	2018	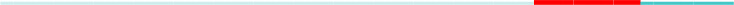
WANDINGER KP	2011	39.52	2011	2019	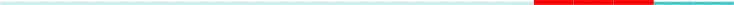
PRÜSS H	2011	38.53	2011	2017	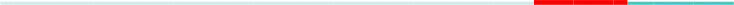
SCHMITT SE	2013	40.89	2013	2019	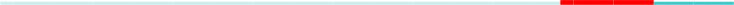
HACOHEN Y	2013	37.35	2014	2017	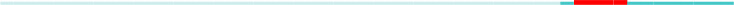
LEYPOLDT F	2013	36.81	2015	2018	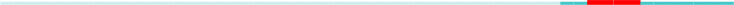
ABBOUD H	2021	108.78	2022	2025	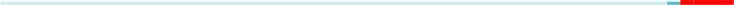
BUDHRAM A	2020	43.85	2022	2025	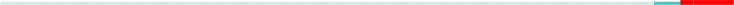
GUASP M	2019	40.33	2022	2025	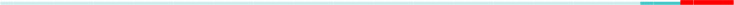
UY CE	2022	45.45	2023	2025	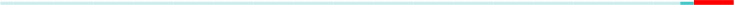
CELLUCCI T	2014	37.64	2023	2025	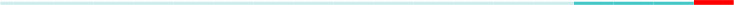
THALER FS	2022	36.87	2023	2025	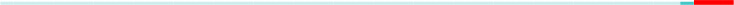

### 3.5 Research hotspots and thematic evolution

**Figure 7:**
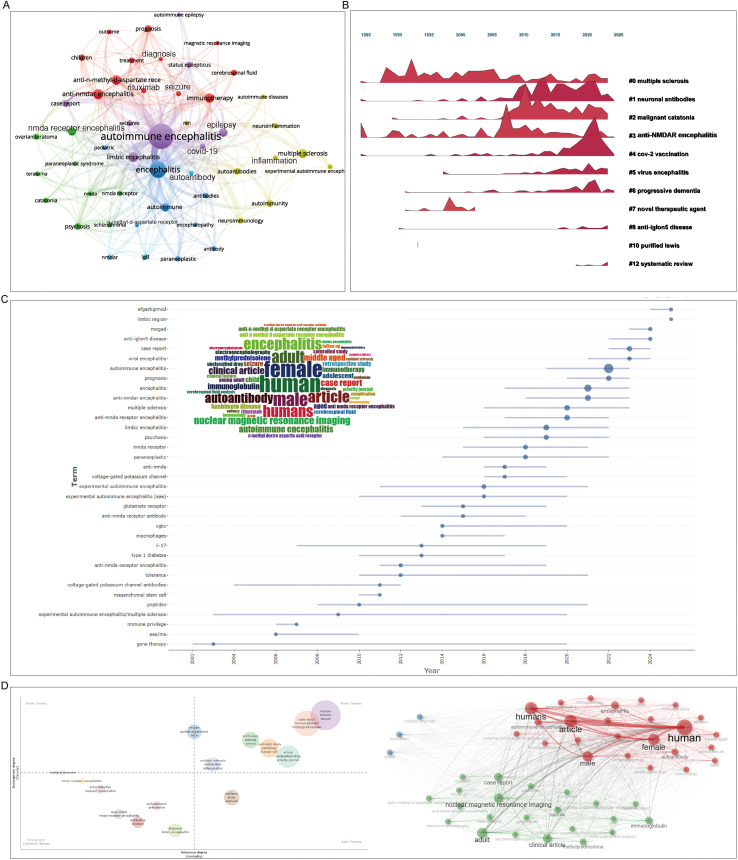
Keywords in AE research. **(A)** Overlay network visualization of author keyword co−occurrence in AE research, created with VOSviewer (weights by documents). **(B)** Keyword mountain plot depicting the temporal evolution of keywords in the AE field, generated by CiteSpace under default settings. **(C)** Word cloud (inset) and trend topic analysis of keywords in AE research, generated by Biblioshiny under default settings. The size of each word in the word cloud represents its frequency of occurrence. **(D)** Thematic map and keyword clustering analysis of AE research, generated by Biblioshiny under default settings.

Analysis of keywords revealed that autoimmune encephalitis and encephalitis occupy the central position in the keyword co-occurrence network. These core terms are strongly linked to keywords in several key thematic categories: etiology-related terms (e.g., NMDA receptor, autoantibody, ovarian teratoma, and their variants); symptoms and related disorders (e.g., epilepsy, multiple sclerosis, autoimmune epilepsy, and their variants); and diagnosis and treatment-related terms (e.g., magnetic resonance imaging, diagnosis, cerebrospinal fluid, rituximab, immunotherapy, treatment). Among these, multiple sclerosis and neuronal antibodies (along with their variants) have received sustained attention in the field (**Figure 7A**).

The keyword evolution plot shows that terms such as multiple sclerosis, anti-NMDAR encephalitis, and neuronal antibodies have remained consistently active between 1985 and 2025. Some terms, like malignant catatonia and viral encephalitis, have shown increasing activity over time. In contrast, other keywords, such as novel therapeutic agent and purified Lewis antigen, had only transient prominence. During the COVID-19 pandemic, a substantial number of studies focused on the relationship between autoimmune encephalitis and COVID-19, resulting in a prominent and highly active cluster of COVID-related keywords in recent years (**Figures 7B, C**). It is noteworthy that keywords such as limbic region, anti-IGLON5 disease, and anti-NMDA receptor encephalitis have attracted more recent attention than terms such as gene therapy and immune privilege (**Figure 7C**).

The thematic evolution map places human-related terms (human, female) and animal-related terms (nonhuman, animals, animal) in the motor themes quadrant, indicating that these are well-developed and central to the field. Also in this quadrant are terms related to publication format (e.g., case report, controlled study, article) and clinical treatments (e.g., immunoglobulin, methylprednisolone), underscoring the field’s dual emphasis on clinical application and investigation of pathological mechanisms. In contrast, the basic themes quadrant includes foundational neurological terms such as epilepsy, brain, and seizures, while diagnosis, limbic encephalitis, antibodies, and auto-antibodies are positioned in the emerging or declining themes quadrant, indicating areas of potential growth or waning interest. The themes co-occurrence network reveals distinct thematic clusters. Specifically, the term “human” is predominantly associated with demographic variables (e.g., “female,” “male”) and general “articles.” In a separate cluster, diagnostic and therapeutic terms such as “nuclear magnetic resonance imaging” and “immunoglobulin” are tightly linked to “case report” and “clinical article.” This clustering pattern likely reflects a substantial proportion of clinical case studies focused on diagnostics and treatments within the analyzed corpus (**Figure 7D**). Further analysis of the Top 23 Terms with the Strongest Citation Bursts revealed that the keyword “ovarian teratoma” exhibited the highest burst strength, reaching 64.14. Correspondingly, the term “young women” also showed a relatively high burst strength. Other keywords such as “anti-nmda receptor” and “anti-nmda receptor encephalitis” similarly demonstrated strong citation bursts, indicating that research in this field has consistently focused on the modulation of the NMDA receptor (**Table 5**).

**Table 5:** Top 23 terms with the strongest citation bursts.

Terms	Year	Strength	Begin	End	1971 - 2025
experimental autoimmune encephalomyelitis	1989	32.85	1989	2015	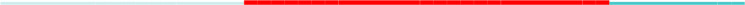
multiple sclerosis	1978	39.46	1995	2011	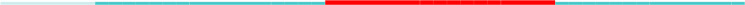
animal model	1997	31.23	1997	2015	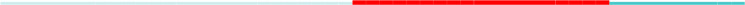
t cells	1990	29.15	2001	2012	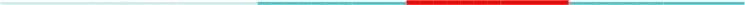
ovarian teratoma	2008	64.14	2008	2017	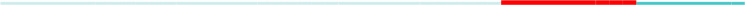
young women	2008	33.86	2008	2017	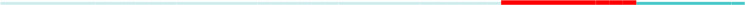
nmda receptor	2008	33.16	2008	2016	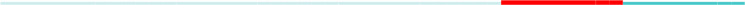
anti-nmdar antibodies	2008	20.34	2008	2017	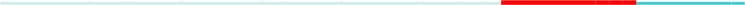
anti-nmda receptor	2008	55.57	2009	2016	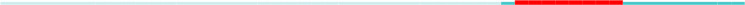
anti-nmda receptor encephalitis	2008	46.57	2009	2015	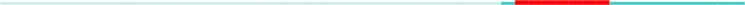
nmda-receptor encephalitis	2009	30.59	2009	2013	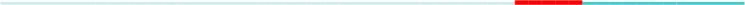
autonomic instability	2009	23.98	2009	2017	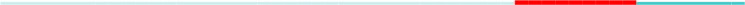
anti-nmda receptor antibodies	2009	16.91	2009	2015	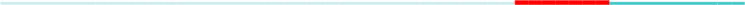
receptor encephalitis	2010	27.51	2010	2017	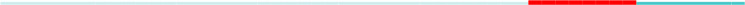
voltage-gated potassium channel	2011	19.61	2011	2018	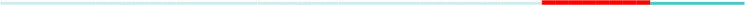
autoimmune disorder	2004	17.15	2011	2018	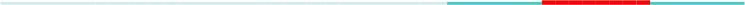
abnormal movements	2004	17.74	2012	2018	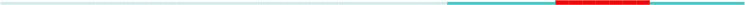
antiepileptic drugs	2015	17.06	2015	2020	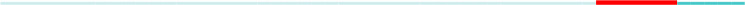
sars-cov-2	2021	20.5	2021	2023	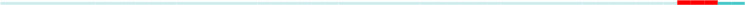
coronavirus disease	2021	17.59	2021	2023	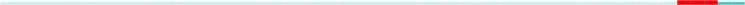
covid-19	2020	27.68	2022	2023	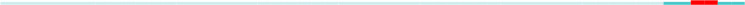
clinical assessment scale	2022	18.41	2023	2025	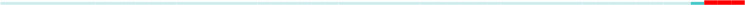
anti-iglon5 disease	2023	16.02	2023	2025	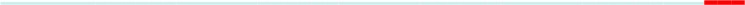

**Figure 8:**
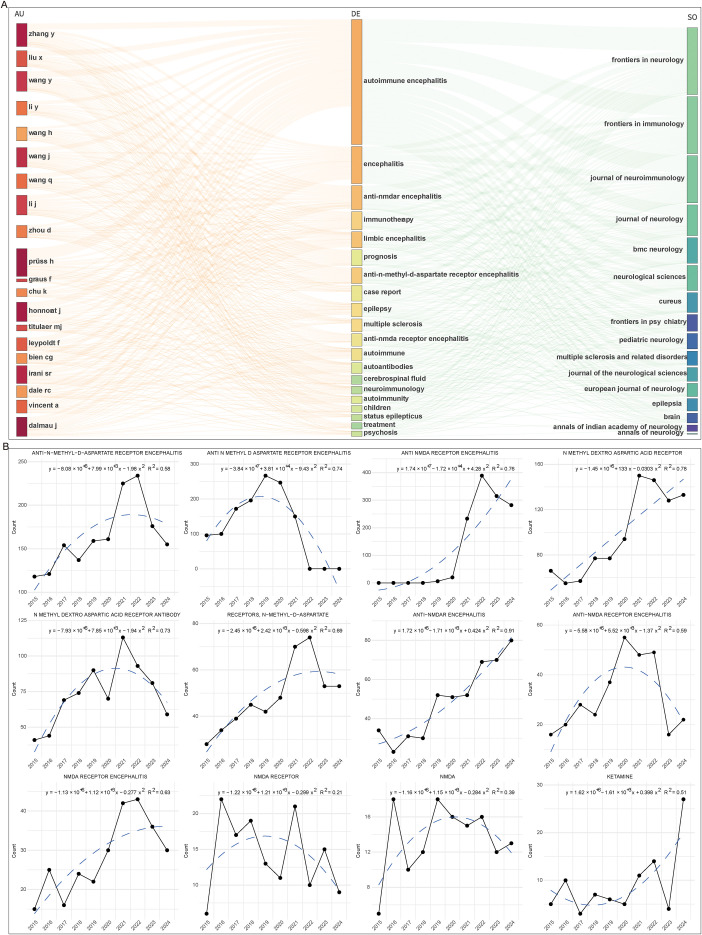
Frontiers in AE research. **(A)** Three-field plot illustrating the relationships among key authors, keywords, and key journals in AE research, generated by Biblioshiny under default settings. **(B)** Temporal trend analysis of keywords specifically involved in Anti-NMDA receptor encephalitis research. Keywords related to Anti-NMDAR AE were extracted from the DE field using a customized R script, and their occurrence trends over time were fitted with growth curve analysis.

We observed in the three-field plot of key authors, keywords, and journals that 19 out of the 20 key authors showed a strong association with Anti-NMDAR autoimmune encephalitis (AE), and related studies were consistently published in key journals (**Figure 8A**). In line with this, an analysis of the top 11 references with the strongest citation bursts revealed that 8 explicitly focused on Anti-NMDAR AE (**Table 6**), underscoring its central role and high impact within the field. The remaining three references were clinically oriented articles addressing the diagnosis of AE and its differentiation from other types of encephalitis (**Table 6**). To further validate this observation, we extracted 12 Anti-NMDAR AE-related keywords from the top 500 keywords and analyzed their research trends from 2015 to 2024. The study reveals that various forms of the term “Anti-N-Methyl-D-Aspartate Receptor Encephalitis”, such as “Anti NMDA Receptor Encephalitis”, “Anti-NMDA Receptor Encephalitis” and “Anti NMDAR Encephalitis”, now appear with higher frequency compared to a decade ago, particularly “Anti NMDA Receptor Encephalitis.” In contrast, the variant “Anti N Methyl D Aspartate Receptor Encephalitis” has shown a decline in usage over the same period. This indicates a growing preference among researchers for the form “Anti NMDA Receptor Encephalitis,” while older naming conventions are gradually being phased out. Overall, the combined frequency of all forms of “Anti-N-Methyl-D-Aspartate Receptor Encephalitis” has increased significantly over the past decade (**Figure 8B**).

**Table 6:** Top 11 References with the Strongest Citation Bursts.

Title	DOI	Year	Citation Strength
Paraneoplastic anti–N-methyl-D-aspartate receptor encephalitis associated with ovarian teratoma	10.1002/ana.21050	2007	75.9
Anti-NMDA receptor encephalitis in Japan-Long-term outcome without tumor removal	10.1212/01.wnl.0000278388.90370.c3	2008	55.9
Anti-NMDA-receptor encephalitis: case series and analysis of the effects of antibodies	10.1016/S1474-4422(08)70224-2	2008	128.6
Anti–N-methyl-D-aspartate receptor (NMDAR) encephalitis in children and adolescents	10.1002/ana.21756	2009	79. 7
N-methyl-d-aspartate antibody encephalitis: temporal progression of clinical and paraclinical observations in a predominantly non-paraneoplastic disorder of both sexes	10.1093/brain/awq113	2010	78.9
Cellular and Synaptic Mechanisms of Anti-NMDA Receptor Encephalitis	10.1523/JNEUROSCI.0167-10.2010	2010	67.9
Treatment and prognostic factors for long-term outcome in patients with anti-NMDA receptor encephalitis: an observational cohort study	10.1016/S1474-4422(12)70310-1	2013	168.5
Antibody titres at diagnosis and during follow-up of anti-NMDA receptor encephalitis: a retrospective study	10.1016/S1474-4422(13)70282-5	2014	61.0
A clinical approach to diagnosis of autoimmune encephalitis	10.1016/S1474-4422(15)00401-9	2016	230.0
Autoimmune encephalitis epidemiology and a comparison to infectious encephalitis	10.1002/ana.25131	2018	53.6
Frequency, symptoms, risk factors, and outcomes of autoimmune encephalitis after herpes simplex encephalitis: a prospective observational study and retrospective analysis	10.1016/S1474-4422(18)30244-8	2018	48.8

## 4 Discussion

### 4.1 Comparison with other studies

Our study provides a comprehensive and updated bibliometric overview of AE research, which distinguishes it from recent similar analyses in several critical aspects (17, 18). First, we extended the temporal scope to cover over five decades (1971-May 2025), capturing the complete evolutionary trajectory from early descriptive reports to the current molecular and therapeutic era, including the pivotal discovery of anti-NMDAR encephalitis. This longer timeframe allows for a more robust analysis of historical trends, inflection points, and sustained thematic evolution compared to studies with shorter intervals (e.g., 1999–2022 or 2005-2023). Second, to ensure a more representative and less biased dataset, we systematically retrieved records from three major databases: PubMed, Web of Science, and Scopus, followed by rigorous deduplication and standardization. This multi-source approach mitigates the database-specific limitations inherent in studies relying solely on the Web of Science Core Collection and enhances the comprehensiveness of the literature sample. Third, beyond common metrics such as publication output and collaboration networks, our analysis delves deeper into thematic evolution, keyword bursts, and the structural integration of emerging methodologies (e.g., AI and structural biology) in recent research frontiers. These layers of analysis offer a more nuanced understanding of how the field is transitioning from syndromic characterization to mechanism-driven therapeutics. Therefore, while acknowledging the valuable contributions of prior bibliometric works, our study delivers a more temporally extensive and analytically detailed mapping of the global AE research landscape, underscoring its necessity for informing future strategic research directions.

### 4.2 Growth trajectory, geographical leadership, and collaborative dynamics

Over the past five decades, autoimmune encephalitis research has undergone a transformative shift. The field experienced limited activity until around 2007, when a pivotal inflection point occurred, coinciding with the seminal discovery of anti-NMDA receptor encephalitis (anti-NMDAR AE). This breakthrough catalyzed an exponential growth phase, with annual publications surpassing 1,600 by 2022, evidenced by high compound annual growth rates and robust exponential fit models (**Figure 2**). This advancement reflects not merely quantitative expansion, but a profound paradigm shifts towards molecular pathogenesis and targeted immunotherapy, fundamentally reshaping the clinical and research landscape of AE.

Geographically, the research output is predominantly concentrated in the United States and China, which have emerged as the leading contributors by volume. The temporal pattern reveals distinct growth models: the United States exhibited sustained, early leadership, whereas China demonstrated a remarkable, albeit later, surge in productivity. However, bibliometric influence, as measured by citation metrics, presents a more nuanced picture. While the U.S. leads in total citations, Spain achieves the highest average citation impact, highlighting that publication volume alone does not equate to scholarly influence. The structure of international collaboration further illuminates the field’s dynamics. The United States serves as the central hub in an extensive global network, while other major producers like China engage in less diversified international partnerships. Emerging research nations show even more limited collaboration. This pattern suggests that while the field is internationalizing, collaboration networks remain uneven, potentially affecting the diffusion of knowledge and innovation. At the institutional level, leadership is held by entities like the University of Pennsylvania, with collaboration clusters forming primarily along transatlantic lines and between Western and leading Chinese institutions. These findings collectively highlight a field driven by a key discovery, marked by concentrated yet asymmetrical geographical productivity, and evolving through complex, hub-centered collaborative networks that may shape future research directions and equity.

### 4.3 Research focus and thematic evolution

Keyword analyses and co-occurrence mapping provided insights into the dominant and evolving research themes. Two primary thematic cores emerged-centered around “human” and “autoimmune encephalitis”-alongside closely related terms such as “rituximab”, “immunotherapy”, “diagnosis”, and “antibody.” A second cluster focused on experimental models and disease severity, while a third included clinical case-related terms such as “fever,” “neuroimaging,” and “tonic-clonic seizure”. The temporal evolution of keywords revealed persistent activity in foundational terms such as “NMDA encephalitis” and “multiple sclerosis” from 1985 to 2025. Meanwhile, emerging topics including “neuronal antibodies”, “malignant catatonia”, and “viral encephalitis” have gained increasing prominence, whereas others like “novel therapeutic agent” and “purified Lewis” only received transient attention. Citation burst analysis further highlighted “anti-NMDA receptor” and “NMDA receptor” as central focal points of intense scholarly interest.

Our analysis confirmed that research on NMDA receptor encephalitis has attracted exceptional attention within the field. This is reflected by the fact that 8 of the 11 most-cited references explicitly addressed Anti-NMDAR AE. Similarly, among the top 100 keywords, seven were directly related to Anti-NMDAR AE, with only one keyword associated with MOG-related encephalitis. Consistent with these findings, the three-field plot linking key authors, keywords, and journals revealed that 19 out of the top 20 core authors had a strong association with Anti-NMDAR AE research.

Building upon the analysis of research focus and thematic evolution, the exceptional scholarly focus on anti-NMDAR AE reflects more than just high publication metrics; our findings suggest it may represent a pivotal shift in neuroimmunology from syndromic description to mechanism-driven disease definition. The consolidation of research around a well-defined antibody-mediated entity has emerged as what could be considered a “paradigm disease”. This paradigm has successfully established a canonical framework-from clinical presentation and diagnostic criteria (antibody testing) to treatment protocols (immunotherapy)-that has fundamentally reshaped the approach to all AE. The intense scrutiny on NMDAR receptors, underscored by the citation bursts, directly bridges basic neuroscience with clinical practice, offering a rare model where molecular structure (the receptor) directly explains core clinical features (psychiatric and neurological symptoms) and predicts treatment response.

However, this concentration of research effort raises the question of whether it risks creating an imbalance. The minimal keyword representation for other types of AE, including Anti-LGI1, CASPR2, or MOG AE, in our data highlights a potential gap, which could indicate that other important AE subtypes are receiving less attention. Whether this reflects actual marginalization or other factors (e.g., later discovery, lower prevalence) requires further investigation.

The emerging thematic shift towards “malignant catatonia” and “viral encephalitis”, alongside the fading transient interest in “novel therapeutic agent”, signals this necessary evolution. It points to a maturation of research questions towards more severe clinical phenotypes, comorbid triggers, and the practical hurdles of drug development. The integration of structural biology and AI, as nascent trends, is poised to be a key enabler in this next phase. These tools can accelerate the mapping of antibody epitopes, predict cross-reactivity, and personalize therapeutic strategies, thereby extending the precision medicine revolution of the entire neuroimmunological landscape. Given the focused research efforts on anti-NMDAR encephalitis, substantial advances have recently been made in elucidating its underlying structural and molecular mechanisms (4, 19). Two independent studies resolved the three-dimensional structure of antibody-NMDA receptor complexes, identifying hotspots for antibody binding. A similar phenomenon has also been observed in structural investigations of anti-GABAR AE (20). Drawing on insights gained from antibody-antigen interaction studies during the SARS-CoV-2 pandemic, researchers have speculated that this epitope represents a hotspot for pathogenic antibody recognition (21). Building on this hypothesis, and leveraging advances in protein structure prediction (22, 23), some investigators have proposed that artificial intelligence could be harnessed to design functional mini-binders targeting this region (24).

**Figure 9:**
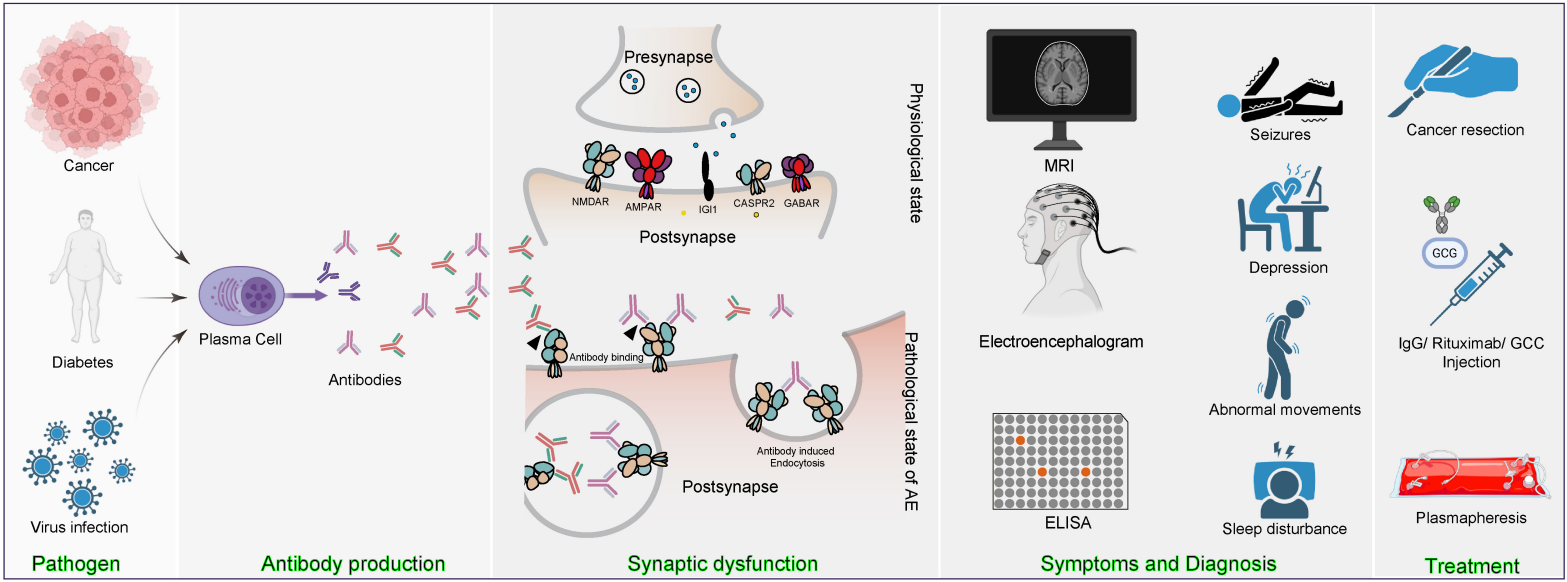
Research trends and frontiers in AE. Major focus areas and frontiers in AE research.

The prominence of numerous clinically related terms among high-burst keywords underscores a strong focus on diagnosis, treatment, and translational applications. This observation is further supported by thematic mapping, which positioned clinical and mechanistic terms such as “human”, “female”, “nonhuman”, “immunoglobulin”, and “methylprednisolone” in the motor themes quadrant, indicating their central and well-developed status within the field. These findings align with clinical observations that a substantial proportion of Anti-NMDAR AE patients are women of reproductive age, many of whom present with associated ovarian teratomas. Neuron-like cells differentiated within these teratomas express high levels of NMDA receptors (25). The resulting antibodies cross the compromised blood-brain barrier, accumulate in the cerebrospinal fluid, and indiscriminately target NMDA receptors on neuronal membranes, leading to receptor internalization via complement-dependent mechanisms. Clinically, this manifests as a range of neurological and psychiatric abnormalities, including seizures, depression, and abnormal movements. First-line therapeutic strategies typically involve tumor resection, corticosteroid administration, total IgG infusion, and Rituximab treatment. Additionally, plasma exchange is often prioritized as it can rapidly reduce antibody titers in both plasma and cerebrospinal fluid, providing prompt symptomatic relief (**Figure 9**).

### 4.4 Clinical orientation and future directions

Our analysis underscores that AE research is intrinsically dual-natured, anchored firmly in both clinical application and mechanistic inquiry. The prominence of terms such as “human”, “female”, “immunoglobulin”, and “methylprednisolone” within the motor themes quadrant reflects a mature, translationally focused knowledge base centered on patient demographics, first-line therapies, and case-based evidence. This aligns with the clinical reality of AE, particularly anti-NMDAR encephalitis, which predominantly affects young women and is frequently managed with immunosuppressive regimens and tumor resection. Conversely, foundational terms like “epilepsy”, “brain”, “limbic encephalitis”, and “autoantibodies” appear in the emerging/declining quadrant, signaling areas where interest is either evolving or holds untapped potential, suggesting that while these concepts are established, they may drive future subfield specialization or novel integrative approaches. The field is now at a transformative stage. While anti-NMDAR encephalitis dominates research, future efforts must broaden to include other subtypes through enhanced clinical and mechanistic studies. Recent advances in other AE subtypes further illustrate this diversification, including structural insights into anti−GABAR encephalitis (20), neural circuit mechanisms in anti−LGI1 encephalitis (26), updated diagnostic criteria for anti−MOG−associated disorders (27), and cellular mechanisms underlying anti−GFAP encephalitis (28).

Looking forward, these findings offer actionable insights for future research priorities and clinical practice: (1) Research prioritization: Funding agencies and research groups should strategically expand efforts beyond anti-NMDAR AE to elucidate the unique pathophysiologies of underrepresented subtypes, leveraging the methodological advances pioneered in anti-NMDAR research; (2) Translational opportunities: The convergence of structural biology and AI highlighted in our analysis points to a roadmap for developing targeted immunotherapies-such as epitope-specific blockers-that could overcome the limitations of current broad-spectrum immunosuppression; (3) Clinical implications: The persistent prominence of clinical keywords (e.g., immunotherapy, rituximab) underscores the need for subtype-specific treatment protocols and biomarkers to guide personalized therapy, moving beyond the current one-size-fits-all approach. By addressing these priorities, the AE field can progress from its current paradigm-disease stage toward a more comprehensive, precision-based framework that improves outcomes for all patients across the autoimmune encephalitis spectrum.

### 4.5 Limitations of the study

While this bibliometric analysis offers a comprehensive overview of the AE research landscape, several inherent limitations must be acknowledged. First, our findings are constrained by the coverage and metadata quality of the three databases used. Despite our multi-source approach, some influential work published in non-indexed sources or regional journals may not be represented. Furthermore, the reliability of bibliometric indicators depends on metadata accuracy; variations in author names, institutional affiliations, and indexing lags, particularly for very recent publications (e.g., 2025), may introduce biases in productivity and collaboration analyses. In addition, the combined use of multiple software tools with proprietary or undisclosed algorithms may introduce inconsistencies in data processing, limiting full transparency and reproducibility. We cannot fully guarantee the reliability of the databases. For instance, in our deduplication process, we validated only a small random sample; thus, we cannot exclude the possibility that duplicate records remain in the dataset. Second, our approach is inherently quantitative and descriptive. While it effectively maps publication trends, networks, and keyword evolution, it does not evaluate the methodological rigor, clinical relevance, or scientific impact of individual studies. Future research integrating qualitative assessments (e.g., systematic reviews or expert consensus) could complement these findings to provide a more nuanced understanding of research quality and translational influence. Third, although our analysis extends to May 24, 2025, the field of AE is evolving rapidly. Emerging disease entities (e.g., anti-IGLON5 disease), novel immunotherapies, and AI-driven discovery tools are continually reshaping the research frontier. Consequently, some recent developments may not be fully captured or proportionally represented in our dataset. Continuous or more frequent updates to such analyses, along with the incorporation of preprint repositories, could help better track real-time shifts in research focus and innovation. Fourth, our search strategy was intentionally designed to capture literature using terminology consistent with the modern conceptualization of autoimmune encephalitis (AE), focusing on antibody-defined subtypes. Consequently, early studies published before 2007, when terms such as “limbic encephalitis” and “paraneoplastic encephalitis” were more commonly used to describe what would now be recognized as AE, may be underrepresented in our dataset. While this approach enhances specificity by avoiding inclusion of non-AE conditions that also involve the limbic system or occur in paraneoplastic contexts, it inevitably introduces a degree of literature loss. This terminology shift should be considered when interpreting long-term publication trends and the apparent acceleration of research activity following the discovery of anti-NMDAR encephalitis. The apparent acceleration of research activity following the discovery of anti-NMDAR encephalitis in 2007 may reflect not only genuine scientific expansion but also the transition to more precise, searchable terminology that better captures the relevant literature.

## 5 Conclusions

This bibliometric analysis comprehensively evaluated the landscape of autoimmune encephalitis (AE) research from 1970s, highlighting key trends in publication output, geographic and institutional contributions, core research themes, and their evolution over time. Rather than merely recapitulating findings, the study situates the observed trends, the exponential growth in publications, the shifting geographic and institutional leadership, and the thematic consolidation around anti-NMDAR encephalitis, within the broader narrative of the field’s evolution. It interprets these patterns as reflections of seminal discoveries, such as the identification of anti-NMDAR AE and highlights how emerging collaboration networks and recent methodological advances (e.g., structural biology and AI) are actively shaping the field’s future trajectory.

## Data Availability

The raw data supporting the conclusions of this article will be made available by the authors, without undue reservation.
